# What Is the Numerical Nature of Pain Relief?

**DOI:** 10.3389/fpain.2021.756680

**Published:** 2021-11-02

**Authors:** Andrew D. Vigotsky, Siddharth R. Tiwari, James W. Griffith, A. Vania Apkarian

**Affiliations:** ^1^Departments of Biomedical Engineering and Statistics, Northwestern University, Evanston, IL, United States; ^2^Center for Translational Pain Research, Feinberg School of Medicine, Northwestern University, Chicago, IL, United States; ^3^Illinois Mathematics and Science Academy, Aurora, IL, United States; ^4^Medical Social Sciences, Feinberg School of Medicine, Northwestern University, Chicago, IL, United States; ^5^Departments of Neuroscience, Anesthesia, and Physical Medicine & Rehabilitation, Feinberg School of Medicine, Northwestern University, Chicago, IL, United States

**Keywords:** pain, clinical trials, treatment effects, statistical models, ANCOVA

## Abstract

Pain relief, or a decrease in self-reported pain intensity, is frequently the primary outcome of pain clinical trials. Investigators commonly report pain relief in one of two ways: using raw units (additive) or using percentage units (multiplicative). However, additive and multiplicative scales have different assumptions and are incompatible with one another. In this work, we describe the assumptions and corollaries of additive and multiplicative models of pain relief to illuminate the issue from statistical and clinical perspectives. First, we explain the math underlying each model and illustrate these points using simulations, for which readers are assumed to have an understanding of linear regression. Next, we connect this math to clinical interpretations, stressing the importance of statistical models that accurately represent the underlying data; for example, how using percent pain relief can mislead clinicians if the data are actually additive. These theoretical discussions are supported by empirical data from four longitudinal studies of patients with subacute and chronic pain. Finally, we discuss self-reported pain intensity as a measurement construct, including its philosophical limitations and how clinical pain differs from acute pain measured during psychophysics experiments. This work has broad implications for clinical pain research, ranging from statistical modeling of trial data to the use of minimal clinically important differences and patient-clinician communication.

## 1. Introduction

Pain is highly prevalent, burdensome, and a common reason for doctor visits (1–4). In an attempt to understand the severity of patients' pain, doctors and researchers ask patients about the intensity of the their pain, requiring patients to condense and transmute their subjective experience to a single number. Despite its abstract and reductionist nature, self-reports of pain intensity are moderately-to-strongly correlated with several patient-reported outcome variables, including quality of life, disability, and more (5, 6). Moreover, self-reports of pain intensity are remarkably easy and inexpensive to collect. These pragmatic and measurement properties make a reduction in self-reported pain, which we define as pain relief, the gold standard for assessing pain improvement.

Clinical studies of pain commonly quantify pain relief as the primary outcome. However, how pain relief is quantified and reported roughly falls into one of two categories: absolute reductions in pain and relative (or percent) reductions in pain. For example, studies that report absolute reduction may state that a drug decreased pain by 2/10 numerical rating scale (NRS) units or 23/100 visual analog scale (VAS) units. Alternatively, studies that report relative reductions may state that pain decreased by 13% units more in the drug group relative to the placebo group. Although both approaches to reporting pain reductions are common, they are conceptually incompatible (unless baseline pain is perfectly homogeneous; see section 2). Their incompatibility begs the question as to whether one approach is more appropriate than the other.

In this paper, we aim to illuminate the issue of absolute vs. relative pain relief^1^. We rely on statistical theory to provide researchers and statistically-minded clinicians with the background necessary to understand these measurement models, for which readers are assumed to be familiar with linear regression. In addition, we empirically analyze four datasets to reinforce and make tangible our conceptual discussion.

## 2. Statistical Background

Whenever one uses data to make a calculation, they are building a model. Every model has assumptions, but still, models should accurately reflect the data they are intending to simplify and thus represent. With regards to modeling pain relief, when reporting absolute changes in pain, one is assuming the process is additive. Alternatively, when reporting percent changes in pain, one is assuming the process is multiplicative. These assumptions have corollaries that *prima facie* may be unclear. In this section, we aim to explain the processes that would generate each of these models and the theoretical implications of these measurement and modeling assumptions.

### 2.1. Additive Model

The additive model and its implications are best understood by defining a *data-generating process*. This involves creating a mathematical model that reflects how one thinks the data are created. Because longitudinal pain relief is of interest, there is commonly at least one pain rating at the beginning of the study (*x*_*i*_) and at least one or more follow-up ratings (*y*_*i*_) for each subject *i*. The additive model of pain relief uses the simple difference between these pain ratings to calculate absolute pain relief (δ_*i*_ = *y*_*i*_−*x*_*i*_), where negative δ_*i*_'s indicate relief and positive δ_*i*_'s indicate worsening of pain. Although straightforward, this is a gross oversimplification.

**Figure 1:**
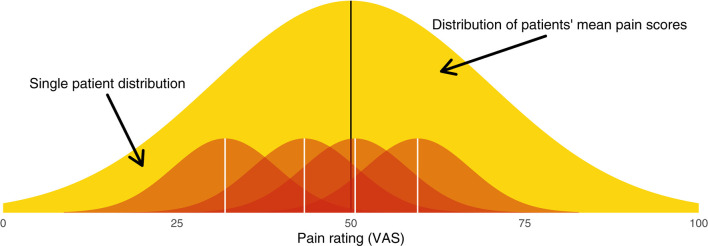
Graphical illustration of the hierarchical model from which patients' pain scores are sampled. The broad yellow (light gray) distribution is the between-patient distribution (level 2), from which each patient's mean pain score is sampled. Each red (dark gray) distribution is a within-patient distribution (level 1), from which single measurements are sampled.

In reality, pain data are messy. For one, between-patient heterogeneity is appreciable—pain ratings at intake will often range from the minimum required for study entry (e.g., 4/10 NRS) to the scale's maximum (e.g., 10/10 NRS). In addition, patients' pain fluctuates from minute-to-minute, hour-to-hour, day-to-day, and so on. To complicate matters further, the process of converting a qualia to a number is undoubtedly fuzzy, meaning the pain ratings themselves will have noise associated with them. Thus, there are two sources of variance to consider: between patients and within patients. These sources of variance can be thought of hierarchically (Figure 1).

Between-patient heterogeneity is a natural place to start. The entire sample of patients will have a mean pain score μ. Each patient's mean at baseline, α_*i*_, will be dispersed around this group mean according to the between-subject variance τ^2^. We can say that patient means are distributed


$$\alpha_{i}\sim N\left(\mu ,\tau^{2}\right).$$

This distribution of patient means is illustrated in yellow in Figure 1.

The notion of within-patient heterogeneity implies there will be variance around each patient's mean pain. When we “sample” a patient's pain rating, we do not observe α_*i*_; rather, we obtain a value α_*i*_ ± σ. These within-patient distributions are illustrated in red in Figure 1. Together, the within- and between-patient models form a hierarchical model (Appendix A1).

**Figure 2:**
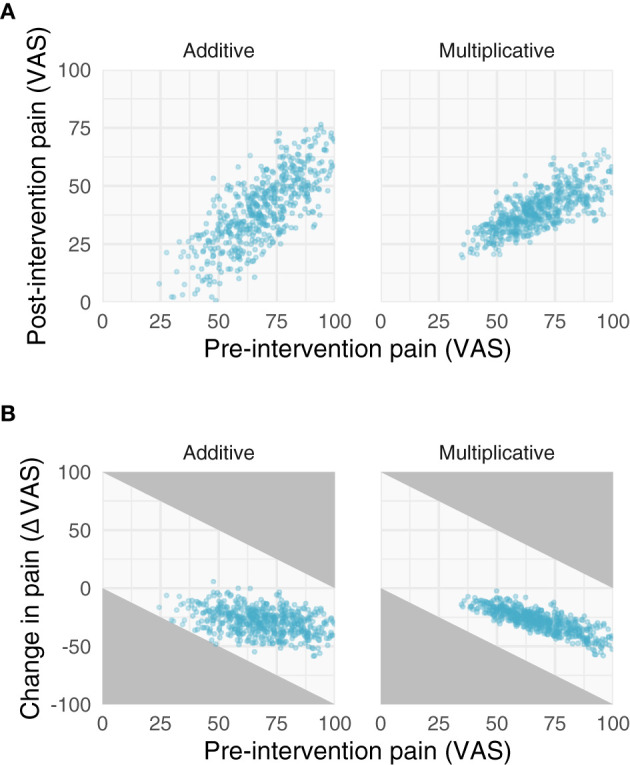
Properties of additive and multiplicative data. We simulated data with additive (left) and multiplicative (right) assumptions. **(A)** Relationships between pre- and post-intervention pain scores when improvements are additive (left) and multiplicative (right). Note the additive post-intervention scores are relatively homoscedastic, while the variance of multiplicative post-intervention scores increases with increasing pre-intervention scores. **(B)** Negative relationships between change scores and pre-intervention scores. Gray areas in **(B)** represent regions where points are not possible due to measurement constraints; that is, because a change score cannot be >|100|.

Because the patient's pre- and post-intervention pain ratings have variability associated with them, the observed difference scores are subject to regression toward the mean (RTM). RTM is a statistical phenomenon whereby higher initial scores are likely to be followed by lower measurements, and similarly, lower initial scores are likely to followed by higher measurements. For example, suppose someone's diastolic blood pressure is normally around 70 mmHg. If a doctor measures that individual's blood pressure and finds it to be 90 mmHg, it is highly probable that the next time it is measured, it will be lower than 90 mmHg. Individuals whose measurements deviate more from their mean will thus appear to undergo greater changes. In the case of a pain study, those who start off with greater pain levels will regress toward the mean, in turn creating larger change scores. This is depicted graphically in Figure 2B, which shows that those who have greater pre-intervention pain scores (*x*-axis) have smaller change scores (*y*-axis). Importantly, this phenomenon is purely statistical and can be explained by the reliability of the measurement.

Measurement reliability is commonly quantified using the intraclass correlation coefficient (ICC). The simplest version of the ICC is the ratio of the between-patient variance to the total variance,


$$\frac{\tau^{2}}{\tau^{2}+\sigma^{2}},$$

where τ^2^ is the between-patient variance and σ^2^ is the within-patient variance. Since σ^2^ defines the variance between individual measurements from a single patient, the ICC can be improved by using the mean of several measurements from a single patient rather than a single measurement. Doing so allows us to substitute σ^2^ with the variance of the sample mean, $\frac{\sigma^{2}}{n}$, giving us an ICC that is a function of the number of data points sampled from each patient,


$$\frac{\tau^{2}}{\tau^{2}+\frac{\sigma^{2}}{n}}.$$

Note, this quantity approaches 1 (perfect reliability) as *n* → ∞.

**Figure 3:**
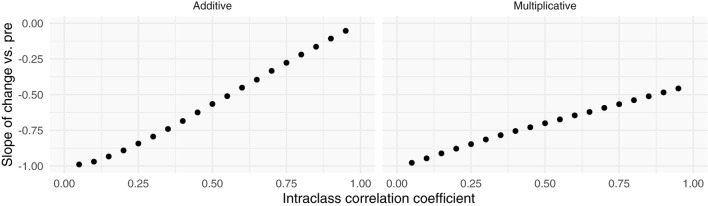
Simulations of additive and multiplicative changes reveal the effect of different intraclass correlation coefficients on the slope between change scores and pre-intervention scores. Additive effects have slopes that trend toward zero with increasing ICC's, while multiplicative effects always have a negative slope no matter their ICC.

Importantly, the above concepts generalize to post-intervention scores as well. If we assume τ^2^ and σ^2^ do not change, and instead, there is a simple shift in mean scores without ceiling and floor effects, then the ICC also defines the Pearson correlation between pre- and post-intervention scores. The Pearson correlation is useful because it gives us direct insight into RTM—the slope between the pre-intervention scores and change scores approaches zero as the correlation between pre- and post-intervention scores approaches 1 (Figure 3).

All of these properties come together and should be considered when statistically modeling pain relief and the effect of an intervention.

### 2.2. Multiplicative Model

The multiplicative model is still mathematically simple but its implications are more complex. If pain relief is multiplicative, then it can be modeled as a relative reduction; i.e., $\phi =\frac{\delta_{i}}{x_{i}}$. This would imply that each person's post-intervention pain (*y*_*i*_) is a fraction of their starting pain (*x*_*i*_); i.e., *y*_*i*_ = (ϕ + 1)*x*_*i*_. However, ratios and relative reductions have unfavorable statistical properties. Instead, it is preferable to work on the log scale (7–9). In particular, recall $\log\frac{y_{i}}{x_{i}}=\log y_{i}-\log x_{i}$, enabling us to linearize the multiplicative process. Similarly, from this, one may realize that it is natural to model multiplicative effects as being generated from *log*-normal distributions rather than normal distributions (Appendix A2).

The implications of the log-normal distribution and its multiplicative properties are shown and described in Figures 2, 3. Note that the multiplicative pain reductions follow a different distribution than additive effects owing to their errors compounding rather than adding. This results in a “fanning” (or heteroscedasticity) of post-intervention scores as a function of greater pre-intervention scores (Figure 2A). This is a hallmark of multiplicative processes that can be evaluated empirically. In addition to this fanning, it is quickly apparent that even with zero measurement error (Figure 3), multiplicative effects can look like RTM since greater pre-intervention scores will result in greater decreases in pain (Figure 2B). However, as opposed to additive processes in which greater pre-intervention scores are attributable to RTM (i.e., measurement error), this relationship is indeed “real” for multiplicative processes.

The multiplicative nature does not only apply to the relationship between pre- and post-intervention pain, but also the effect of a treatment. This is described in further detail in the next subsection.

### 2.3. Statistical Models of Pain Relief

Randomized controlled clinical trials aim to compare pain between two groups. To do so, investigators commonly compare the absolute or percent pain relief itself (e.g., a *t*-test on the change scores). However, such analyses are ill-conceived. Instead, especially for studies that record one or few follow-up measures (as opposed to time-series), it is recommended that the data-generating process be modeled using an analysis of covariance (ANCOVA) with pre-intervention scores as a covariate (8, 10). The reasons for this are manifold:

1. The response variable in a statistical model should be the result of an experiment. Because patients enter studies with their baseline score, it is not the result of the experiment so it should not be treated as a dependent variable (e.g., like in a group × time analysis of variance).
2. Accounting for RTM. Instead of a group × time analysis of variance, one could perform a simple *t*-test on the change scores. However, such an analysis ignores RTM, and, especially in the case of baseline imbalances, can produce biased estimates. ANCOVA can adjust for such effects.
3. Improving statistical efficiency. ANCOVA has greater statistical efficiency, resulting in greater power and more precise intervals.
4. Post-intervention scores are arguably more interesting than change scores. Patients must live with the pain following the intervention, not the change in pain. However, regressing post-intervention pain *or* change in pain produces the same group effect (8).

These statistical and philosophical advantages are well-established in the biostatistics literature (8, 10–14). Note, the benefits of ANCOVA primarily apply to randomized studies, as ANCOVA may produce biased estimates in non-randomized studies depending on the allocation mechanism (15).

For the additive case, the ANCOVA model takes the form


$$y_{i}=\beta_{0}+\beta_{1}x_{i}+\beta_{2}g_{i}+\epsilon_{i},$$

where $\epsilon_{i}\sim N\left(0,\sigma^{2}\right)$ and *g*_*i*_ is dummy-coded for group (e.g., 0 = placebo and 1 = drug). β_2_ is the effect of interest: the average difference in post-intervention pain scores between groups after adjusting for pre-intervention scores. β_1_ will typically be < 1, indicative of RTM, and the intercept may be nonsensical unless *x*_*i*_ is mean-centered. Of course, like any regression, one can add more covariates, especially those with prognostic value, which will further increase statistical efficiency.

The ANCOVA can also be generalized to the multiplicative case. Since multiplicative effects can be linearized by taking the log-transform, we can write the model as


(1) $$y_{i}=B_{0}\cdot x_{i}^{\beta_{1}}\cdot B_{2}^{g_{i}}\cdot E_{i}$$

(2) $$\;=\exp\left\{\beta_{0}+\beta_{1}\log x_{i}+\beta_{2}g_{i}+\epsilon_{i}\right\}$$

(3) $$\Rightarrow \log y_{i}=\beta_{0}+\beta_{1}\log x_{i}+\beta_{2}g_{i}+\epsilon_{i}.$$

This model reveals a few things. First, in (1), residuals will compound with increasing values of the predicted *y*_*i*_ (i.e., ŷ_*i*_). Indeed, this is consistent with what we observed in the simulations above, so this functional form can capture the compounding error. Second, in (3), both *y*_*i*_ and *x*_*i*_ are logged, so when β_1_ = 1, it is equivalent to modeling the percent change; however, when β_1_ ≠ 1, there is a scaling to account for nonlinearities and RTM. Finally, *B*_2_ is a multiplicative effect: when *B*_2_ = 1, both groups are expected to have the same post-intervention score for a given pre-intervention score; when *B*_2_ > 1, the experimental group is expected to have a greater post-intervention score for a given pre-intervention score; and so on. Since we are fitting β_2_ rather than *B*_2_, the fit coefficient will be on the log scale, so exponentiating the coefficient will make it more interpretable despite the log scale having nicer mathematical properties. Note, even this multiplicative ANCOVA is more efficient than analyzing percent changes (12).

## 3. Empirical Data

As a proof of principle, we assessed the properties of four separate datasets. Two of the datasets were collected in patients with subacute back pain and the other two consist of patients with chronic back pain. Ideally, data are analyzed using intention-to-treat. However, here, we included individuals for whom we had enough ratings to complete our analyses as the data are being used for illustrative purposes and we are not looking to draw inferences.

### 3.1. Datasets

#### 3.1.1. Placebo I (Chronic Back Pain)

##### 3.1.1.1. Overview

The purpose of this study was to investigate factors associated with placebo analgesia in chronic pain patients (16). This was the first trial designed to study chronic pain patients receiving placebo vs. no treatment. The total duration of the study lasted ~15 months. Protocol and informed consent forms were approved by Northwestern University IRB and the study was conducted at Northwestern University (Chicago, IL, USA).

##### 3.1.1.2. Participants

To meet inclusion criteria, individuals had to be 18 years or older with a history of lower back pain for at least 6 months. This pain should have been neuropathic (radiculopathy confirmed by physical examination was required), with no evidence of additional comorbid chronic pain, neurological, or psychiatric conditions. Individuals had to agree to stop any concomitant pain medications and had to be able to use a smartphone or computer to monitor pain twice a day. Additionally, the enrolled patients had to report a pain level of at least 5/10 during the screening interview, and their averaged pain level from the smartphone app needed to be higher than 4/10 during the baseline rating period before they were randomized into a treatment group. A total of 82 patients were randomized. Here, we include 18 participants from the no treatment group and 42 participants from the placebo group for whom we had complete rating data [cf. Supplementary Figure 1 in (16)].

##### 3.1.1.3. Pain Data

Data were collected using a custom pain rating phone app through which patients could rate their pain (0–10 NRS). Patients were asked to enter their pain 2 times/day over the course of the entire study. For the purposes of demonstration, here we averaged pain ratings within a single day.

#### 3.1.2. Placebo II (Chronic Back Pain)

##### 3.1.2.1. Overview

The purpose of this study was to validate a prognostic model for classifying chronic pain patients based on their predicted improvement with placebo (17). Protocol and informed consent forms were approved by Northwestern University IRB and the study was conducted at Northwestern University (Chicago IL, USA).

##### 3.1.2.2. Participants

Individuals with chronic low back pain were recruited for this study. Patients must have had low back pain for at least 6 months, with or without symptoms of radiculopathy, a minimum VAS score of 5/10 at the screening visit and a minimum average pain of 4/10 over a 2-week period prior to their first visit. A total of 94 patients were randomized to no treatment, placebo, or naproxen. Here, we include 12 participants from the no treatment group, 33 participants from the placebo group, and 35 participants from the naproxen group for whom we had complete rating data [cf. Figure 1 in (17)].

##### 3.1.2.3. Pain Data

Data were collected using a custom pain rating phone app through which patients could rate their pain (0–10 NRS), as in Placebo I. Patients were asked to enter their pain 2 times/day over the course of the entire study. For the purposes of demonstration, here we averaged pain ratings within a single day.

#### 3.1.3. Levodopa Trial (Subacute Back Pain)

##### 3.1.3.1. Overview

The purpose of this trial was to investigate whether levodopa (l-DOPA) can block patients' transition from subacute to chronic back pain (18). This 24-week double-blind parallel group randomized controlled trial was conducted at Northwestern University (Chicago, IL, USA). Protocol and informed consent form were approved by Northwestern University IRB as well as NIDCR/NIH. All enrolled participants provided written informed consent. The trial was registered on ClinicalTrials.gov, under registry NCT01951105.

##### 3.1.3.2. Participants

Individuals with a recent onset of low back pain were recruited. Criteria for enrollment included history of low back pain with a duration between 4 and 20 weeks with signs and symptoms of radiculopathy and average reported pain intensity > 4 (on an NRS scale from 0 to 10) on the week before baseline assessments and the week preceding treatment start. Participants were randomized to one of three groups: no treatment (completed *n* = 10), naproxen + placebo (*n* = 28), naproxen + l-DOPA/c-DOPA (*n* = 21). Here, we will use data from 47 patients who had complete rating data (naproxen + placebo = 27; naproxen + l-DOPA/c-DOPA = 20) [cf. Figure 1B in (18)].

##### 3.1.3.3. Pain Data

Data were collected using a custom pain rating phone app through which patients could rate their pain (0–10 NRS). Patients were asked to enter their pain 3 times/day over the course of the entire study (28 weeks). For the purposes of demonstration, here we averaged pain ratings within a single day.

#### 3.1.4. Prospective Cohort (Subacute Back Pain)

##### 3.1.4.1. Overview

The purpose of this study was to identify predictive biomarkers to identify individuals who will vs. will not recover from subacute back pain (19). Protocol and informed consent forms were approved by Northwestern University IRB as well as NIDCR/NIH, and the study was conducted at Northwestern University (Chicago, IL, USA). All enrolled participants provided written informed consent. All participants were right-handed and were diagnosed by a clinician for back pain. An additional list of criteria was imposed including: pain intensity > 40/100 on the visual analog scale (VAS) and duration < 16 weeks.

##### 3.1.4.2. Participants

Eighty individuals with a recent onset (within 16 weeks) of lower back pain and an average reported pain intensity > 40/100 (on the VAS) who completed at least three follow-up visits (i.e., 30 weeks following the initial visit).

##### 3.1.4.3. Pain Data

Data were collected at five separate visits using the short form of the McGill Pain Questionnaire (MPQ). The computed sensory and affective scores from the MPQ for each visit are used as individual pain scores for each subject.

### 3.2. Data Properties

**Figure 4:**
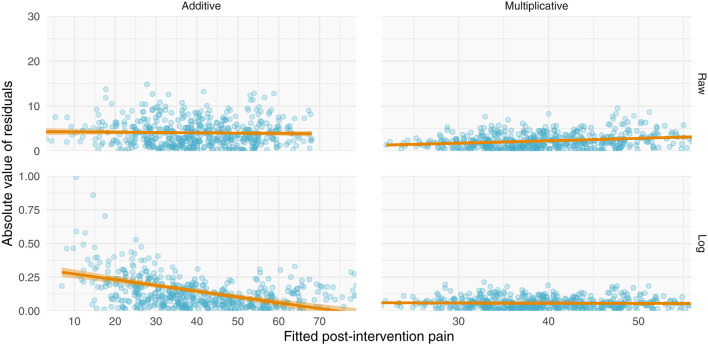
Simulations of additive and multiplicative changes reveal differential residual behavior for raw and log-transformed ANCOVA models. **(Left)** data generated with have an additive structure have homoscedastic residuals when fit with a standard ANCOVA (top) but heteroscedastic residuals when fit with a log-transformed ANCOVA (bottom). **(Right)** data generated with a multiplicative structure have heteroscedastic residuals when fit on their raw scale (top) but homoscedastic residuals when log-transformed (bottom).

To evaluate whether each dataset was more compatible with an additive or multiplicative process, we conducted the same analyses from the Statistical Background section (Figures 2–4) on these data. In particular, we investigated properties of the raw and log-transformed data, in addition to the properties of ANCOVAs fit to the data. To do so, all data were converted to a 0–100 scale. Before log-transforming, we added 1 to the raw scores to avoid log(0) = NaN. In doing so, we demonstrate how the aforementioned principles apply to real data.

**Figure 5:**
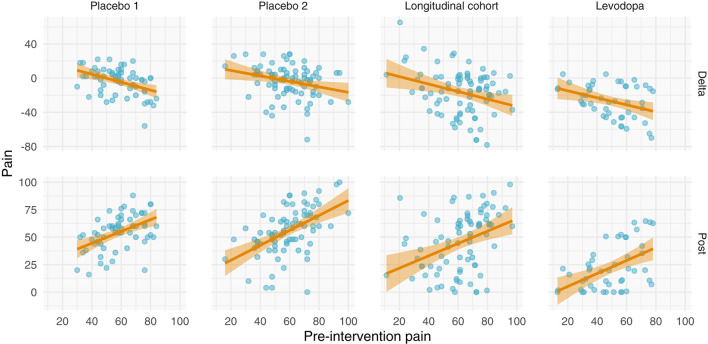
Relationships between pre-intervention scores and change scores (top) and post-intervention scores (bottom). **(Top)** Relationship between pre-intervention scores and change scores. Note that most of the studies have a negative relationship. This could be explained by regression toward the mean or multiplicative effects, in addition to ceiling/floor effects. **(Bottom)** Relationship between pre-intervention and post-intervention pain scores across all studies. Each study shows a positive relationship between pre- and post-intervention scores; however, the Levodopa study appears to have greater variance in post-intervention scores with greater pre-intervention scores.

All datasets have positive relationships between pre- and post-intervention scores (Figure 5). Interestingly and in contrast to the other studies, the variance of the post-intervention scores in the levodopa trial appears to increase with greater pre-intervention scores, consistent with a multiplicative effect. Finally, with the exception of the prospective cohort study, there are negative relationships between changes in pain and pre-intervention scores. These negative relationships may be explained by multiplicative effects or RTM. Further examination is needed to ascertain the nature of these data.

**Figure 6:**
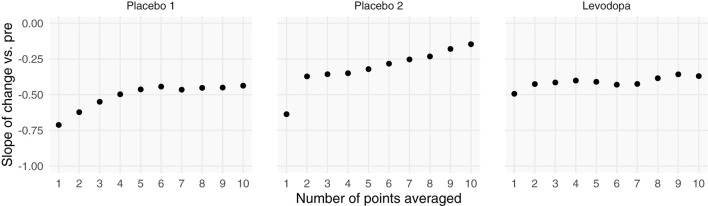
Increasing the number of points used for each patient's pre- and post-intervention scores increases the slope between change scores and pre-intervention scores. Each patient's pre- and post-intervention scores were calculated using the mean of *x* points. By averaging over more points, we should increase the intraclass correlation coefficient. Negative slopes between change scores and pre-intervention scores are indicative of one of two things: (1) regression toward the mean or (2) multiplicative effects. In the datasets that show evidence of being additive, we see marked increases in slopes, indicating that we are decreasing regression toward the mean by including more points. However, because the Levodopa Trial displays multiplicative properties, it is only minimally affected by adding more points.

Including more points in the calculation of pre-intervention and post-intervention scores increases the ICC, thereby increasing the reliability and decreasing the effect of RTM (Figure 3). Since three of the four datasets contained ecological momentary assessments of pain, we were able to sample and average more than one point from the beginning and end of each study. We averaged an increasing number of a pre- and post-intervention points and recalculated the slope between change score and pre-intervention score (i.e., plot from Figure 5, top). If the slopes strongly trend toward zero by increasing the number of points, this indicates that the data have additive properties. Slopes that stay negative regardless of increasing reliability (number of points) indicate that the data may be multiplicative. For the studies included in this analysis (Placebo I, Placebo II, Levodopa Trial), Placebo I and Placebo II's slopes have slight upward trends: as the number of points in the calculation of pre-intervention and post-intervention scores increases, the negative slope due to RTM increases. In contrast, the Levodopa trial's negative slopes remain stable (Figure 6). This again hints at the notion that the levodopa trial's data may be multiplicative, while Placebo I and Placebo II may be additive.

**Figure 7:**
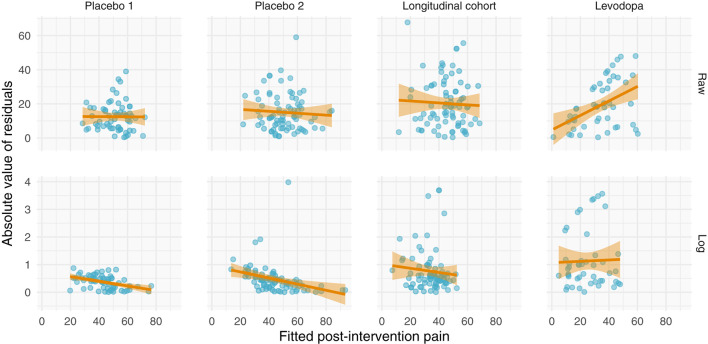
Absolute values of residuals from additive ANCOVA models. We fit an ANCOVA to each dataset using pre-intervention score and group membership as covariates. From these models, we plotted the absolute values of the residuals as a function of the fitted value. Additive models should be homoscedastic, meaning the magnitudes of the residuals do not change as a function of the response variable. However, multiplicative models have compounding error, such that if you fit them using an additive model, greater predicted values will be associated with larger magnitudes of residual error. Placebo I, Placebo II, and the Prospective Cohort study all exhibit features of additive data. However, the Levodopa Trial exhibits multiplicative properties, as evidenced by the increasing error residual magnitude with increasing fitted values.

Perhaps the most direct assessment of additive vs. multiplicative properties is to model the data and assess the model fits. When assessing and utilizing a model, one should ensure that the model's assumptions are met and that the model captures salient features of the data. Because multiplicative data-generating processes lead to compounding residuals, we can observe these effects when fitting ANCOVAs. In Figure 7, we focus specifically on the variance observed in Figure 5, illustrating the relationship between fitted values (using the ANCOVA models from Figure 5) and the absolute value of the residuals. As shown in Figure 2, multiplicative relationships possess higher variance as pre-intervention scores increase, compared to additive relationships which are homoscedastic. For this reason, we should observe a null correlation between fitted values and absolute residual error for data that have exhibited additive properties (Placebo I, Placebo II, Prospective Cohort) thus far, and observe a positive correlation between fitted values and absolute residual error for data that have exhibited multiplicative properties (Levodopa Trial). As predicted, the Placebo I, Placebo II, and Prospective Cohort data all display this additive quality, as their residual error does not increase as fitted values increase. In contrast, the Levodopa Trial data display multiplicative properties, as its residual error increases as fitted values increase. The description and analyses of these data can be seen below (Figure 7).

From these plots, it is clear that the Placebo I, Placebo II, Prospective Cohort demonstrate additive properties while the Levodopa Trial demonstrates multiplicative properties. An understanding of these concepts and model assumptions have real implications. In Table 1, we include the average absolute (additive) and log-transformed (multiplicative) change in pain scores for each dataset. As an example, the effect of naproxen relative to no treatment in Placebo II is −15 (−27, −3) for the additive model but 0.7 (0.4, 1.1) for the multiplicative model. The 95% CI is much wider for the multiplicative model since it is misspecified, which in turn may lead an investigator or clinician to be less certain conclusions about the treatment effect.

**Table 1:** Additive and multiplicative effects by dataset.

**Dataset**	**Additive model (NRS), $\hat{\beta }$ (CI_**95*%***_)**	**Multiplicative model (AU), $\hat{\beta }$ (CI_**95*%***_)**
Placebo I	−3 (−12, 5)	0.9 (0.8, 1.1)
Placebo II	Placebo: −9 (−21, 4) Naproxen: −15 (−27, −3)	Placebo: 0.8 (0.5, 1.3) Naproxen: 0.7 (0.4, 1.1)
Levodopa trial	4 (−7, 15)	1.5 (0.7, 3.3)

*All effects were modeled using ANCOVA with pre-intervention scores as a covariate. Multiplicative effects use the log-transformed scores and represent the exponentiated coefficients which can be interpreted as the relative effect of treatment group vs. the control group [e.g., post-intervention pain in the placebo group (Placebo I) will be 90% of the post-intervention pain in the no treatment group]*.

## 4. Discussion

Pain relief is a ubiquitous clinical trial outcome with direct treatment implications. Treatments that yield appreciable pain relief will be employed in the clinic, and findings from these trials may be communicated to patients. However, if data from trials are not properly modeled, then the resulting treatment effects may be both biased and highly variable, which in turn may mislead researchers, clinicians, and patients. In this theory-based paper, we have emphasized the difference between additive and multiplicative treatment effects from mathematical, statistical, and empirical perspectives. It is clear that the assumptions behind these effects are not interchangeable and thus should be more thoughtfully considered when planning and analyzing clinical trial data. Moreover, how pain relief is conceptualized will propagate into the interpretation of effects, which we briefly discuss herein.

### 4.1. Minimal Clinically Important Differences

Pain intensity ratings can be difficult to interpret—they are a reductionist, unidimensional measurement intended to capture a single aspect of a private, complex, incommunicable experience (20, 21). To help make sense of improvements, researchers and clinicians commonly rely on minimal clinically important differences (MCID). In clinical pain research, MCIDs are commonly derived by mapping changes in pain ratings onto a different scale, such as global impression of change (22). For example, what absolute change in NRS and relative change in NRS correspond to “much improved”? This mapping is then commonly used as a guidepost for interpreting other studies, and in some cases, individual patient changes (23).

Although commonly derived and used without justification, absolute and relative MCIDs are not interchangeable since they are mathematically incompatible with one another. Suppose patient A starts with an 8/10 pain and patient B starts with a 4/10 pain. If the treatment has an additive effect, both patients may improve by 2/10, but this would result in markedly different percent reductions: 25 and 50% for patients A and B, respectively. Farrar et al. (22) suggest that an MCID for pain relief is 2/10 NRS or 30%; here, these would yield two different conclusions since both patients achieved a 2/10 decrease but only one patient achieved a 30% decrease. Much attention has been and continues to be given to both additive and multiplicative MCIDs without considering the conceptual difference between the two. This conceptual incompatibility needs to be reconciled if MCIDs are to be used in a meaningful way. However, there are also larger issues that warrant addressing.

Across studies and ignoring the numerical nature of treatment effects, MCIDs have a linear relationship with baseline pain ratings, with an *x*-intercept corresponding to roughly 30/100 and a slope of 1 (i.e., MCID ≈ baseline − 30) (24). This relationship calls into question both absolute and relative MCIDs. If absolute MCIDs were valid, then we would expect the MCID to be constant across all baseline pain scores. If relative MCIDs were valid, then we would expect a *y*-intercept of 0 and a slope equal to the MCID. Rather, this relationship suggests MCIDs are more compatible with a post-intervention pain rather than a change score, and this post-intervention pain is equal to 30/100. In other words, the MCID is the change in pain needed to obtain a 30/100. If true, this would be consistent with the idea that it is a patient's pain, not change in pain, that is important.

More generally, MCIDs arguably represent a conflation of constructs. MCIDs typically involve dichotomizing a measurement by mapping it onto some other measurement using some loss function—a form of “dichotomania” (25). For example, researchers may threshold and dichotomize changes in VAS into improvement vs. non-improvement using the global impression of change scale (22). This dichotomization of pain scores is then applied to other studies. Yet, such an approach is curious—it implies we are actually interested in global impression of change but use pain scores as a noisy proxy. If a researcher is interested in global impression of change, they should measure global impression of change as an outcome in their sample. Further, the ontological basis for dichotomous change scores is arguably ill-conceived. The insipid use of MCIDs in pain research and practice deserves greater scrutiny. From this perspective, it has been argued that greater context is needed in deriving metrics of clinical importance (26, 27) for which decision theory may provide a rigorous foundation.

In addition to using MCIDs for interpreting findings, researchers have used MCIDs for “responder analysis.” For example, a researcher may split patients into groups of “responders” and “non-responders” based on whether their change in pain exceeded the MCID [see section 4.5 in (23)]. However, such analyses have undesirable properties on both the individual and group levels. On the individual level, inferences cannot be made regarding response magnitude for several reasons. First, individual counterfactuals are not observed in parallel group trials; for example, we do not know what an individual's pain would have been had they been randomized to the placebo group instead of the drug group. An individual's observed improvement or worsening may have been due to the intervention or alternatively, RTM, natural history, or some other unmeasured, stochastic process. Second, the individual may not reliably attain the same improvement each time the trial is performed; for example, 60% of individuals may respond 100% of the time *or* 100% of individuals may respond 60% of the time (or some mixture of the two). Third, this dichotomization assumes an improvement of, say, 30 and 100% are equivalent, and similarly, that an improvement of 29 and 0% are equivalent (assuming MCID = 30%) by treating improvements as a binary step function rather than continuous—such an assumption strains credulity. These issues have been previously discussed in great detail (28–31). On the group level, dichotomizing individual responses turns each patient's pain improvement into a 0 (“non-responder”) or 1 (“responder”), which discards information and, in turn, markedly decreases statistical efficiency and power (32, 33). Thus, the dichotomization of improvements is arguably unethical since it discards information, effectively decreasing the sample size (32) and, in turn, the ability to quantify (or rule out) meaningful intervention effects. Rather than being treated as an analytical tool, MCIDs are perhaps better viewed from an interpretive and decision-making perspective.

Notwithstanding MCID's limitations, it is perhaps most useful at the planning stage of clinical research. A clinically important difference is just one approach to justifying an effect size of interest for a study (34), which may be used for sample size calculations or stopping rules in adaptive trials. However, beyond planning, dichotomizing trial and especially individual patient outcomes using an MCID is a questionable practice that commonly ignores context and variability (9).

### 4.2. Scale Assumptions

Psychological measurement scales have a rich history across the fields of psychometrics and psychophysics (35). Anchors determine the extremes within which a participant must rate their experience, ultimately constraining the measurement construct and how accurately participants understand what they are rating (36). Bounded by these anchors, the measurements themselves can be on one of a number of scales: nominal, ordinal, interval, ratio, and absolute. Nominal scales assume a one-to-one mapping between the desired quantity *x*′ and the measured quantity *x*; ordinal scales assume a monotonic mapping; interval scales assume an affine mapping (*x*′ = *ax* + *b*); ratio scales assume a linear mapping with an absolute zero (*x*′ = *ax*); and absolute scales assume a perfect mapping (*x*′ = *x*) (37). Several renowned psychophysicists have argued—not without criticism (38, 39)—that perceptual ratings are or can easily be converted to ratio scale (35, 37). Importantly, the additive and multiplicative models rely on interval and ratio assumptions, respectively. Thus, the validity of these assumptions for clinical pain must be considered.

The numerical nature of clinical pain is an open, controversial, and perhaps unanswerable question. Early psychophysics work argues that VAS and NRS pain scales are ratio for both experimental and clinical pain. Price et al. (40) used cross-modality matching to argue that clinical pain, like heat pain, is a ratio scale. However, by mapping clinical pain onto heat pain, this finding is arguably tautological—they assessed whether clinical pain-matched heat pain follows the same power law as heat pain. Others have used item-response theory to argue that pain ratings are ordinal scale (nonlinear) rather than ratio or interval scale (41). Since the authors used unidimensional measures and a Rasch model, this conclusion is based on stationarity assumptions and ratings' reliability, which are not necessary conditions for interval or ratio scales. Although the perceptual ratings from psychophysics are undoubtedly related to clinical pain, assessing the measurement properties of clinical pain is much more complex since we cannot precisely control the sensory input. Thus, clinical pain measurement scale assumptions arguably cannot be rigorously evaluated, reinforcing that they are indeed assumptions. However, the strength of assumption varies, with interval scales (additive) having weaker assumptions than ratio scales (multiplicative). The assumptions a researcher makes directly affects the model they should choose.

### 4.3. Statistical Modeling and Applications

The choice of a statistical model can greatly affect the inferences drawn from the same dataset. Here, we observed that applying a multiplicative model to a dataset that exhibits additive properties can create wide CIs, making it difficult to interpret the results of an experiment (Table 1). This is consistent with the idea that a properly specified model will be more statistically efficient (12), and perhaps most importantly, it will better represent the underlying data.

We presented two ways of modeling data: additively and multiplicatively. Both rely on ANCOVA, with the former using raw pain scores and the latter using log-transformed pain scores. These models have different assumptions about the underlying data and, as a result, have different interpretations. If authors feel the linearity and ratio assumptions are too strict, there are other models that can be used; e.g., ordinal regression and semiparametric (or nonparametric) ANCOVA (42), in addition to intensive longitudinal and time-series analysis (43). Indeed, there are good examples in the pain literature of ANCOVA-type models being implemented with more complicated data structures [e.g., multiple study endpoints, see (44)]. In any case, researchers should be aware of the assumptions of their statistical models of the properties of their data, and of course, researchers are encouraged to collaborate with statisticians (45).

### 4.4. Recommendations

We have clearly demonstrated the mathematical, conceptual, and interpretive differences between additive and multiplicative effects. From this explication, there are tangible takeaways and recommendations for clinical researchers. Specifically, we suggest that researchers include and consider the following:

1. When deciding which metric to use—absolute pain decreases or percent pain decreases—use the data as a guide unless there is a principled reason to choose one or the other. Since it is unclear what influences the presence of additive or multiplicative characteristics in pain data, it is safer to use the metric that accurately represents the properties of the data. Table 2 summarizes the differences between additive and multiplicative properties. In time, we may develop a better understanding of pain conditions and improvements such that more general recommendations can be provided. We view this data-driven approach as being no different than checking statistical model assumptions.
2. When reporting descriptive statistics, use the arithmetic mean to calculate between-subject (average) intervention for additive data; conversely, use geometric mean for multiplicative data.
3. Ensure that patients' pre-intervention scores are heterogeneous for drawing conclusions about the nature of the data. By including a wide range of pre-intervention scores, it makes the additive or multiplicative properties more apparent. If the data are not heterogeneous, false conclusions may be made about the data's additive or multiplicative properties.

**Table 2:** Hallmarks of additive and multiplicative effects.

**Plot**	**Additive**	**Multiplicative**
Slope of change score vs. pre-intervention score (*y*) vs. number of points (*x*)	Slopes approach zero as the number of points utilized in calculating pre- and post-intervention pain scores increases by increasing ICC (Figure 3, left).	Slopes increase minimally with increasing number of points (Figure 3, right).
Absolute value of residuals (*y*) vs. fitted values (*x*)	No relationship between absolute residual error and fitted (post-intervention) values.	Positive, heteroscedastic relationship between absolute residual error and fitted (post-intervention) values.

## 5. Conclusion

The properties of changes in self-reported pain are commonly implicitly assumed to be additive, multiplicative, or are conflated. Ignoring the properties of pain relief can result in model mis-specification, in turn leading to bias and statistical inefficiency. These errors further propagate into metrics such as minimal clinically important differences. We contend that more attention should be paid to the statistical properties of pain relief to ensure model assumptions are met. By paying closer attention to these properties, we can gain more insight from and make better use of data from pain clinical trials.

## Data Availability Statement

Publicly available datasets were analyzed in this study. This data can be found at: OpenPain.org.

## Ethics Statement

The studies involving human participants were reviewed and approved by Northwestern University. The patients/participants provided their written informed consent to participate in this study.

## Author Contributions

AV, ST, and AVA conceptualized the paper. AV drafted the paper. AV and ST produced the figures. AV, ST, JG, and AVA read, provided feedback on, approved the final version of, and agree to be accountable for the contents of the manuscript. All authors contributed to the article and approved the submitted version.

## Funding

This work was funded by the National Institutes of Health (1P50DA044121-01A1). This material was based upon work supported by the National Science Foundation Graduate Research Fellowship under Grant No. DGE-1324585.

## Conflict of Interest

The authors declare that the research was conducted in the absence of any commercial or financial relationships that could be construed as a potential conflict of interest.

## Publisher's Note

All claims expressed in this article are solely those of the authors and do not necessarily represent those of their affiliated organizations, or those of the publisher, the editors and the reviewers. Any product that may be evaluated in this article, or claim that may be made by its manufacturer, is not guaranteed or endorsed by the publisher.

## Footnotes

^1^For simplicity, herein, we will refer to self-reported pain intensity simply as pain.

## Appendix

### A. Data Generating Processes

#### A.1. Additive Model

The additive model can be conceptualized hierarchically. First, we will assume each individual's average pre-intervention pain, α_*i*_ for patient *i*, is sampled from a larger population,

$$\alpha_{i}\sim N\left(\mu ,\tau^{2}\right).$$

Since α_*i*_ represents an individuals *average* pre-intervention pain, it is a latent construct and ignores measurement error and natural pain variability; for example, minute-to-minute, hour-to-hour, and day-to-day fluctuations in pain intensity. In actuality, an experiment will sample an individual's pain ratings and will be affected by measurement error. Thus, a given measurement of a patient's pre-intervention pain will be

$$x_{ij}=\alpha_{i}+\epsilon_{ij},$$

where $\epsilon_{ij}\sim N\left(0,\sigma^{2}\right)$ for measurement *j* from patient *i*, assuming all patients have the same within-patient variability (Figure 1). If we sample and average *n* measurements from patient *i*, we obtain

$$x_{i\cdot }\sim N\left(\alpha_{i},\frac{\sigma^{2}}{n}\right).$$

Similarly, assuming homogeneous improvement and treatment effects, the average post-intervention pain rating for patient *i* is

$$y_{i\cdot }\sim N\left(\alpha_{i}+\delta +\theta g_{i},\frac{\sigma^{2}}{n}\right),$$

where δ is the improvement in the control group, θ the treatment effect of interest, and *g*_*i*_ is a dummy variable for group (0 = control; 1 = intervention). Without loss of generality via the additive assumption of treatment effects, we will ignore treatment groups (θ) to simplify the problem and describe the properties of these distributions, giving us the simplified post-intervention pain distribution

$$y_{i\cdot }\sim N\left(\alpha_{i}+\delta ,\frac{\sigma^{2}}{n}\right).$$

For both the pre and post model, the intraclass correlation coefficient (ICC) is

$$ICC=\frac{\tau^{2}}{\tau^{2}+\frac{\sigma^{2}}{n}},$$

which is also the correlation between pre- and post-intervention scores. Luckily, ICC is sensitive to the number of data points from which each patient's pre- and post-intervention mean pain scores are calculated,

$$\underset{n\to \infty }{\lim}\frac{\sigma^{2}}{n}=0\Rightarrow \underset{n\to \infty }{\lim}\frac{\tau^{2}}{\tau^{2}+\frac{\sigma^{2}}{n}}=1.$$

With more data points, the slope attributable to RTM disappears. Since the ICC is equivalent to a Pearson's *r* in this case, we can write the joint pre-post distribution of averaged pain scores can be written as a multivariate normal,

$$\left(\begin{array}{c} x_{i\cdot }\\ y_{i\cdot } \end{array}\right)\sim N\left(\left(\begin{array}{c} \mu \\ \mu +\delta \end{array}\right),\left[\begin{array}{cc} \tau^{2}+\frac{\sigma^{2}}{n} & \;\tau^{2}\\ \tau^{2} & \;\tau^{2}+\frac{\sigma^{2}}{n} \end{array}\right]\right).$$

#### A.2. Multiplicative Model

The log-normal distribution is an exponentiated normal distribution, meaning the log of the log-normal distribution is a normal distribution. Therefore, we have

$$\log\alpha_{i}\sim N\left(\log\left(\frac{\mu^{2}}{\sqrt{\mu^{2}+\tau^{2}}}\right),\log\left(1+\frac{\tau^{2}}{\mu^{2}}\right)\right).$$

And like the additive case, a single pre-intervention score *j* for patient *i* can be described as being centered around their individual mean,

$$\log x_{ij}\sim N\left(\log\alpha_{i},\frac{\sigma }{\mu }\right).$$

Similarly, a patient's post-intervention pain is scaled rather than shifted by the change in pain, δ,

$$\log y_{ij}\sim N\left(\log\alpha_{i}+\log\left(1+\frac{\delta }{\mu }\right),\frac{\sigma }{\mu }\right).$$
